# Extracellular ATP Functions as a Metabolic Lineage Selection Signal That Stabilizes Tc9 Cells During Adoptive T Cell Therapy

**DOI:** 10.3390/ijms27073169

**Published:** 2026-03-31

**Authors:** Jie Ren, Zhengrong Gong, Yutong Zhong, Ruipei Xiao, Khadija Urooj, Yuan Gao, Enguang Bi, Handuo Wang

**Affiliations:** 1 Department of Biochemistry and Molecular Biology, School of Basic Medical Sciences, Southern Medical University, Guangzhou 510515, China; 2 Department of Urology, Zhujiang Hospital, Southern Medical University, Guangzhou 510515, China

**Keywords:** ATP, IL-9^+^ CD8^+^ T cells, adoptive T cell therapy (ACT), resident memory T cells (TRM), cancer immunotherapy, mitochondria

## Abstract

Adoptive T cell therapy (ACT) remains limited in solid tumors by poor T cell persistence within the metabolically hostile tumor microenvironment (TME). Although IL-9-producing CD8^+^ T cells (Tc9) consistently demonstrate superior antitumor efficacy compared with conventional Tc1 cells, the selective pressures that shape their functional advantage remain unclear. Here, we show that effective ACT-mediated tumor control is accompanied by a marked increase in intratumoral extracellular ATP (eATP), representing a common metabolic consequence of tumor cell destruction. Despite comparable ATP accumulation following Tc1 or Tc9 treatment, these subsets exhibit strikingly distinct responses to ATP stress. Tc1 cells are highly susceptible to ATP-induced apoptosis, whereas Tc9 cells display intrinsic resistance, resulting in superior in vivo persistence. Mechanistically, Tc9 cells actively convert ATP signaling into enhanced mitochondrial fitness, characterized by increased oxidative phosphorylation and spare respiratory capacity. ATP exposure further drives Tc9 cells toward a tissue-resident memory (TRM) phenotype through activation of the TGF-β signaling axis. Transcriptomic and molecular analyses reveal that purinergic signaling pathways, including elevated expression of the ATP receptor P2RX7, are intrinsically enriched in Tc9 cells and are further amplified upon ATP stimulation. Collectively, our findings identify extracellular ATP as a metabolic lineage selection signal in ACT, demonstrating that ATP stress preferentially stabilizes metabolically resilient Tc9 cells by linking purinergic sensing to mitochondrial remodeling and TRM programming, thereby providing a conceptual basis for enhancing the persistence and efficacy of engineered T cell therapies in solid tumors.

## 1. Introduction

Adoptive T cell therapy (ACT) has revolutionized the treatment of hematologic malignancies and is increasingly being explored for solid tumors [1,2,3]. However, the efficacy of ACT in solid tumors remains limited, largely due to the metabolically and immunologically hostile tumor microenvironment (TME), which constrains T cell survival, persistence, and functional stability [4,5,6]. While significant attention has focused on immune checkpoint pathways and suppressive cytokines, less is understood about how metabolic stress signals generated during tumor destruction shape T cell fate within the TME.

Tumor cell killing is not a metabolically neutral process. Cytotoxic destruction of tumor cells leads to the release of intracellular metabolites and danger-associated molecular patterns into the extracellular space, thereby reshaping the metabolic landscape of the TME [7,8,9,10,11]. Among these signals, extracellular ATP (eATP) represents a highly abundant and dynamic metabolite released from stressed or dying tumor cells [5,12,13,14,15,16]. Traditionally, eATP has been characterized as a damage-associated molecular pattern capable of activating inflammasome signaling [17,18]. In the context of cancer, however, elevated eATP levels are frequently associated with immunosuppression, either through hydrolysis into adenosine via CD39/CD73-expressing cells or through direct induction of T cell apoptosis at high concentrations [5,12,13,19,20]. Purinergic signaling, particularly through the P2RX7 receptor, has been linked to mitochondrial remodeling, and the acquisition of tissue-resident memory (TRM) phenotypes [21,22,23]. Moreover, TRM formation is closely associated with TGF-β signaling and represents a key determinant of long-term T cell persistence within solid tumors [24,25]. These observations suggest that eATP functions as a potent metabolic stress signal within tumors. Yet whether ATP acts uniformly on all T cell subsets—or instead serves as a selective pressure shaping T cell lineage fitness—remains unclear.

CD8^+^ cytotoxic T lymphocytes (CTLs) constitute the primary effector population in ACT, but they exhibit substantial functional heterogeneity. Conventional Tc1 cells, characterized by IFN-γ and TNF-α production, mediate direct tumor cytotoxicity but are prone to exhaustion and loss of persistence within the TME [26,27,28]. In contrast, IL-9–producing Tc9 cells display enhanced longevity, resistance to terminal exhaustion, and superior antitumor efficacy across multiple tumor models [27,29]. Despite these observations, the environmental factors that preferentially stabilize Tc9 cells over Tc1 cells during ACT remain poorly defined. In particular, how distinct CTL subsets interpret and respond to ATP-rich tumor environments has not been investigated. Notably, Tc9 cells are polarized in the presence of IL-4 and TGF-β, which may endow them with an intrinsic predisposition for tissue residency and metabolic resilience [30]. Whether this intrinsic programming enables Tc9 cells, which exhibit superior antitumor function, to uniquely sense and adapt to eATP stress remains a critical unresolved question.

Here, we propose that extracellular ATP functions not merely as a cytotoxic stressor but as a metabolic lineage selection signal during ACT. We demonstrate that effective tumor control is accompanied by robust ATP accumulation within the TME. While ATP induces apoptosis in Tc1 cells, Tc9 cells exhibit intrinsic resistance and actively convert ATP signaling into enhanced mitochondrial fitness and tissue-resident memory (TRM) programming through enriched purinergic signaling pathways. These findings establish ATP stress as an instructive metabolic cue that preferentially stabilizes metabolically resilient Tc9 cells, thereby coupling tumor destruction to lineage adaptation and persistence. Our study provides a conceptual framework for leveraging metabolic signals to optimize engineered T cell therapies for solid tumors.

## 2. Results

### 2.1. ATP Release by Tumor Cells Is Elevated During ACT-Mediated Tumor Control

**Figure 1:**
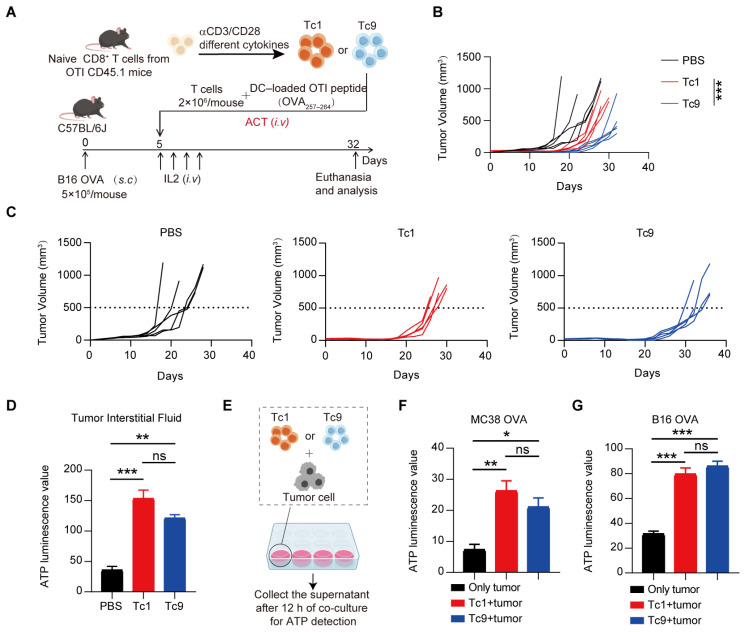
eATP release by tumor cells is elevated during effective tumor control. (**A**) Schematic of the experimental design for ACT. Naive CD8^+^ T cells isolated from OTI CD45.1 mice were polarized under different cytokine conditions (Tc1 vs. Tc9) in vitro. C57BL/6J mice bearing established B16-OVA tumors (subcutaneously injected at 5 × 10^5^ cells/mouse) received an intravenous injection of 2 × 10^6^ polarized T cells. Mice were euthanized and analyzed 32 days after T cell transfer (*n* = 5 mice per group). (**B**) Tumor growth curves in mice treated with PBS, Tc1 cells, or Tc9 cells. (**C**) Tumor growth kinetics for individual mice across PBS, Tc1, and Tc9 treatment groups. (**D**) ATP luminescence levels in tumor interstitial fluid from mice treated with PBS, Tc1 or Tc9 cells (Normalization was performed based on the tumor weight). (**E**) Experimental design for detecting ATP levels in the supernatant after 12 h of in vitro co-culture of tumor cells with OTI Tc1 or Tc9 cells. (**F**) The ATP luminescence value in the cell supernatant of OT1 Tc1 and Tc9 cells co cultured with MC38 OVA for 12 h. (**G**) The ATP luminescence value in the cell supernatant of OT1 Tc1 and Tc9 cells co cultured with B16 OVA for 12 h (*n* = 3 biologically independent replicates, each derived from naive CD8^+^ T cells isolated from individual mouse spleens). Data are presented as mean ± SEM. * *p* < 0.05, ** *p* < 0.01, *** *p* < 0.001, ns, not significant.

As reported previously [27,28], Tc9 cells consistently mediated superior tumor control compared with Tc1 cells in our ACT model (Figure 1A–C), indicating enhanced tumoricidal activity in vivo. Because cytotoxic tumor destruction is intrinsically coupled to the release of intracellular metabolites, we next examined whether effective ACT reshapes the metabolic landscape of the tumor microenvironment, particularly with respect to extracellular ATP (eATP).

Notably, intratumoral ATP levels were markedly increased following both Tc1 and Tc9 treatment (Figure 1D). Importantly, despite the superior tumor control achieved by Tc9 cells, ATP accumulation occurred to a comparable extent in both treatment groups. To validate this observation, we performed in vitro co-culture assays and quantified ATP in the supernatant. Consistent with the in vivo findings, both Tc1 and Tc9 cells induced robust ATP release from tumor cells, with no significant difference between the two subsets (Figure 1E–G).

These results indicate that ATP accumulation represents a general metabolic consequence of T cell–mediated tumor killing rather than a reflection of differential therapeutic efficacy. Thus, elevated eATP appears to be an intrinsic feature of successful ACT and may function as a feedback metabolic signal capable of shaping T cell fate within the tumor microenvironment.

### 2.2. Tc9 Cells Exhibit a Survival Advantage Under High-ATP Stress Through Resistance to ATP-Induced Apoptosis

**Figure 2:**
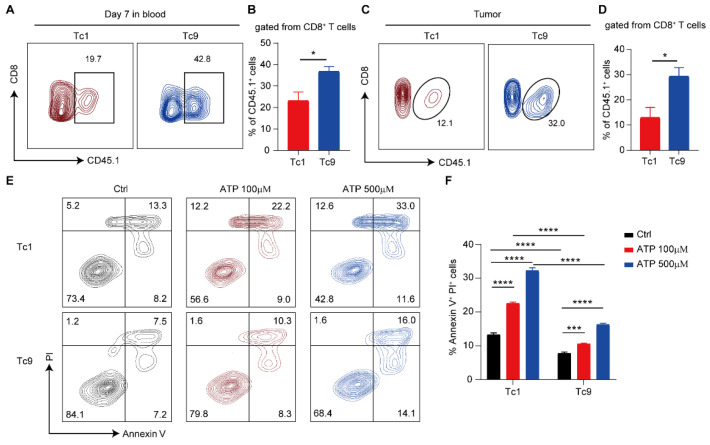
Tc9 cell resistance to ATP-induced apoptosis in vitro. (**A**) Representative flow cytometry plots showing the percentage of transferred Tc1 and Tc9 cells in the blood at 7 days post-transfer (gated from CD8^+^ T cells). (**B**) Quantification of the proportion of transferred Tc1 and Tc9 cells for each group in the blood (gated from CD8^+^ T cells). (**C**) Representative flow cytometry plots showing the infiltration of transferred Tc1 and Tc9 cells in tumors (gated from CD8^+^ T cells). (**D**) Quantification of the proportion of transferred Tc1 and Tc9 cells in tumors (gated from CD8^+^ T cells) (*n* = 5 mice per group). (**E**) Representative flow cytometry plots showing Annexin V and PI staining of Tc1 and Tc9 cells on day 10 treated with ATP at the indicated concentrations. (**F**) Statistical analysis of the percentage of apoptotic cells (Annexin V^+^ PI^+^) in each treatment group (*n* = 3 biologically independent replicates, each derived from naive CD8^+^ T cells isolated from individual mouse spleens). Data are presented as mean ± SEM. * *p* < 0.05, *** *p* < 0.001, **** *p* < 0.0001.

Given that ATP accumulation represents a common metabolic consequence of ACT-mediated tumor killing, we next asked whether Tc1 and Tc9 cells differentially respond to ATP stress. In vivo analysis revealed that transferred Tc9 cells were present at significantly higher frequencies than Tc1 cells in both peripheral blood and tumor tissues (Figure 2A–D), indicating superior persistence within the host.

Because elevated extracellular ATP has been reported to induce T cell apoptosis, we hypothesized that differential sensitivity to ATP may underlie this persistence advantage. To directly test this possibility, Tc1 and Tc9 cells were exposed to increasing concentrations of ATP in vitro, and apoptosis was quantified. We observed that Tc1 cells were highly susceptible to ATP-induced apoptosis, displaying a dose-dependent increase in Annexin V^+^ PI^+^ cells as ATP concentrations rose (Figure 2E,F). In contrast, Tc9 cells exhibited marked resistance to ATP-induced cell death, with only a modest increase in apoptosis even at high ATP concentrations (500 μM) (Figure 2E,F).

Collectively, these findings suggest that Tc9 cells possess an intrinsic capacity to withstand ATP-induced cytotoxic stress, which likely contributes to their superior persistence within the high-ATP tumor microenvironment.

### 2.3. ATP Enhances Mitochondrial Fitness in Tc9 Cells Under Metabolic Stress

Given that Tc9 cells exhibit resistance to ATP-induced apoptosis, we next sought to determine whether ATP stress differentially impacts mitochondrial fitness in Tc1 and Tc9 cells. Because mitochondrial integrity and oxidative metabolism are central to T cell persistence and memory formation, we evaluated key parameters of mitochondrial function following ATP exposure.

**Figure 3:**
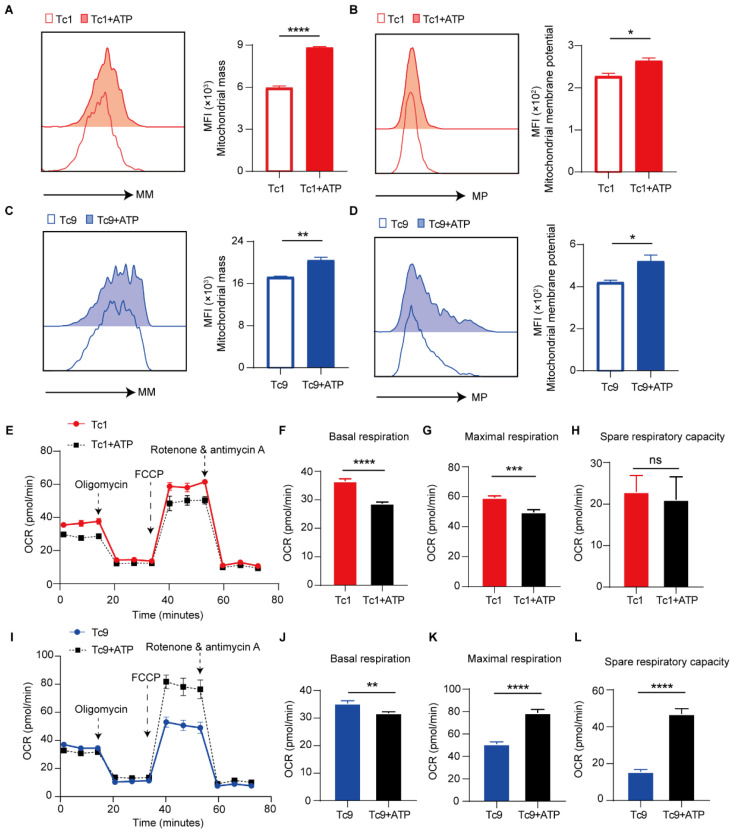
Mitochondrial metabolic remodeling in Tc1 and Tc9 cells under ATP treatment. (**A**) Representative plots and quantification showing the mean fluorescence intensity (MFI) of mitochondrial mass (MM, staining by MitoTracker) in Tc1 cells on day5 treated with ATP and untreated (Ctrl). (**B**) Representative plots and quantification showing the MFI of mitochondrial membrane potential (MP, staining by TMRM) in Tc1 cells on day 5 treated with ATP and Ctrl. (**C**) Representative plots and quantification showing the MFI of MM in Tc9 cells on day5 treated with ATP and Ctrl. (**D**) Representative plots and quantification showing the MFI of MP in Tc9 cells on day 5 treated with ATP and Ctrl. (**E**) The OCR of Tc1 cells on day 10 cultured alone or co-cultured with ATP during mitochondrial stress test. (**F**–**H**) Quantification of respiratory parameters derived from OCR, (**F**) basal respiration, (**G**) maximal respiration, and (**H**) spare respiratory capacity. (**I**) The OCR of Tc9 cells on day 10 cultured alone or co-cultured with ATP during mitochondrial stress test. (**J**–**L**) Quantification of respiratory parameters derived from OCR, (**J**) basal respiration, (**K**) maximal respiration, and (**L**) spare respiratory capacity (*n* = 3 biologically independent replicates, each derived from naive CD8^+^ T cells isolated from individual mouse spleens). Data are mean ± SEM. * *p* < 0.05, ** *p* < 0.01, *** *p* < 0.001, **** *p* < 0.0001, ns, not significant.

Upon treatment with ATP or the P2X7 agonist BzATP, both Tc1 and Tc9 cells showed increased mitochondrial mass (MM) and mitochondrial membrane potential (MMP), indicating that ATP signaling actively modulates mitochondrial dynamics at the early differentiation stage (Figure 3A–D and Supplementary Figure S1). Notably, functional metabolic assessment using Seahorse analysis revealed a distinct advantage between Tc1 and Tc9 cells on day 10, at which point the cells had undergone two rounds of stimulation, more closely resembling their physiological state in vivo. In Tc1 cells, basal respiration and maximal respiration were significantly reduced (Figure 3E–H). However, ATP-treated Tc9 cells displayed significantly enhanced oxidative phosphorylation, characterized by increased maximal respiration, and spare respiratory capacity (Figure 3I–L), and these parameters are all key determinants of cell survival.

These findings indicate that, rather than merely surviving ATP stress, Tc9 cells actively convert purinergic signaling into enhanced mitochondrial fitness. This metabolic adaptation may provide a bioenergetic foundation for their sustained persistence within the high-ATP tumor microenvironment.

### 2.4. ATP Preferentially Programs Tc9 Cells Toward a Tissue-Resident Memory Fate

**Figure 4:**
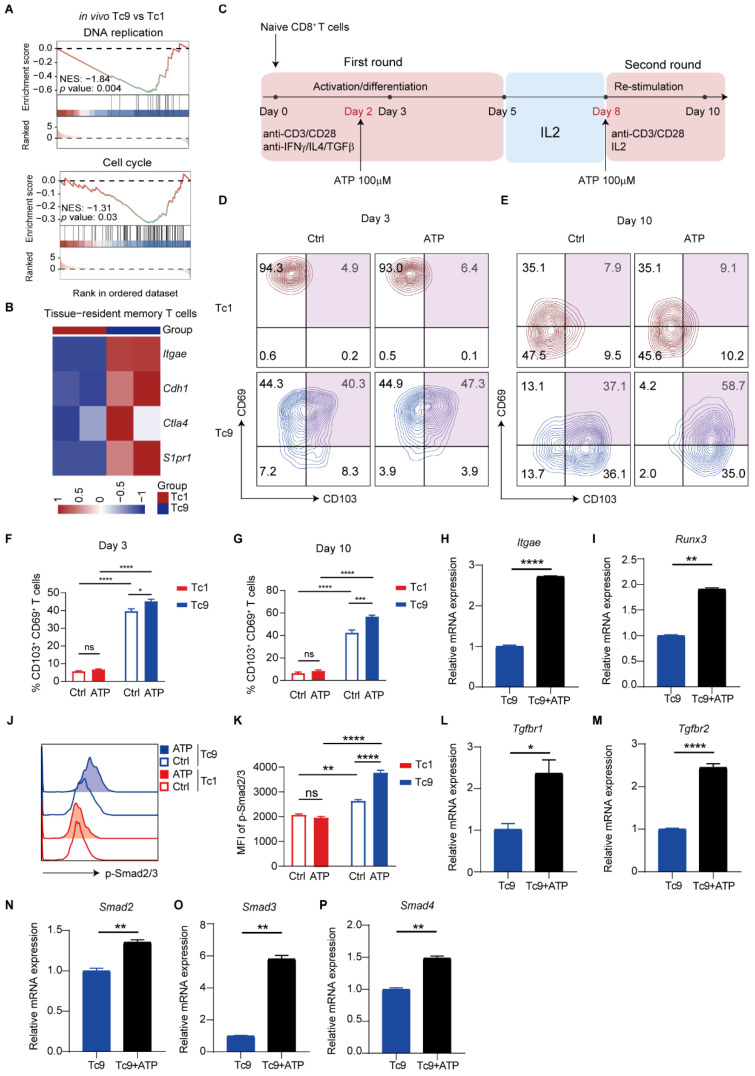
ATP activates the TGF-β signaling pathway and promotes the TRM phenotype in Tc9 cells in a subset-specific manner. (**A**) Gene Set Enrichment Analysis (GSEA) plots demonstrating significant downregulation of the DNA replication and Cell cycle gene sets in tumor-infiltrating Tc9 cells compared to Tc1 cells. (**B**) Heatmap of RNA-seq data showing the expression levels of TRM cell signature genes in endogenously sorted Tc1 and Tc9 populations in tumor [31]. (**C**) Schematic of the in vitro differentiation and stimulation protocol for generating Tc1 and Tc9 cells from naive CD8^+^ T cells (Tc1 cells were induced with IL-2 alone). (**D**,**E**) Representative flow cytometry plots showing the co-expression of the surface markers CD69 and CD103 on in vitro-differentiated Tc9 cells at early (day 3) and late (day 10) time points after initiation of differentiation treated with ATP or not. (**F**,**G**) Quantification of the percentage of CD69^+^ CD103^+^ T cells at the indicated time points in different groups. (**H**,**I**) Quantitative PCR analysis of mRNA expression of TRM-associated genes (*Itgae* and *Runx3*) in Tc9 cells cultured with or without ATP. (**J**,**K**) Representative plots and quantification showing the MFI of p-Smad2/3 in Tc1 and Tc9 cells on day 10 treated with ATP and Ctrl. (**L**–**P**) Quantitative PCR analysis of mRNA expression of TGF-β signaling pathway related genes (*Tgfbr1*, *Tgfbr2*, *Smad2*, *Smad3* and *Smad4*) in Tc9 cells on day 10 (*n* = 3 biologically independent replicates, each derived from naive CD8^+^ T cells isolated from individual mouse spleens). Data are presented as mean ± SEM. * *p* < 0.05, ** *p* < 0.01, *** *p* < 0.001, **** *p* < 0.0001.

Because mitochondrial fitness is tightly linked to T cell differentiation and long-term memory programming, we next investigated whether ATP-induced metabolic remodeling influences Tc9 lineage fate. Transcriptomic analysis of tumor-infiltrating cells revealed that, compared with Tc1 cells, Tc9 cells exhibited downregulation of DNA replication and cell cycle pathways in vivo (Figure 4A), consistent with a transition toward a quiescent or memory-like state. Moreover, Tc9 cells displayed elevated expression of canonical tissue-resident memory (TRM) markers, including *Itgae* (CD103) (Figure 4B).

To recapitulate this differentiation trajectory in vitro, we performed sequential restimulation assays (Figure 4C). Tc9 cells progressively acquired a TRM phenotype, as evidenced by increased CD69 and CD103 expression along the differentiation stages treated with ATP (Figure 4D–G). Importantly, ATP treatment further amplified the TRM program. Exposure of Tc9 cells to ATP significantly upregulated TRM-associated transcriptional regulators, including *Itgae* and *Runx3* (Figure 4H,I). However, Tc1 cells did not exhibit a TRM phenotype at any stage of differentiation, regardless of ATP treatment (Figure 4D–G). Given that TGF-β signaling is a central driver of both Tc9 differentiation and TRM formation, we examined components of this pathway following ATP treatment. ATP exposure enhanced protein expression of p-Smad2/3 in Tc9 cells, whereas no significant changes were observed in Tc1 cells (Figure 4J,K). At the mRNA level, Tc9 cells exhibited elevated expression of *Tgfbr1*, *Tgfbr2*, and downstream Smad signaling molecules *(Smad2/3/4*), indicating activation of the TGF-β axis in Tc9 cells (Figure 4L–P).

Collectively, these results demonstrate that ATP not only enhances mitochondrial fitness but also actively programs Tc9 cells toward a tissue-resident memory phenotype. Thus, ATP functions as a metabolic instructive signal, coupling bioenergetic adaptation to lineage stabilization within the tumor microenvironment.

### 2.5. Purinergic Signaling Is Intrinsically Enriched in Tc9 Cells and Underlies Their Selective Responsiveness to ATP

Although ATP promotes mitochondrial fitness and TRM differentiation in Tc9 cells, we next sought to determine why Tc9 cells exhibit heightened sensitivity to purinergic cues while remaining resistant to ATP-induced apoptosis. We hypothesized that intrinsic differences in purinergic signaling machinery may account for this selective responsiveness.

**Figure 5:**
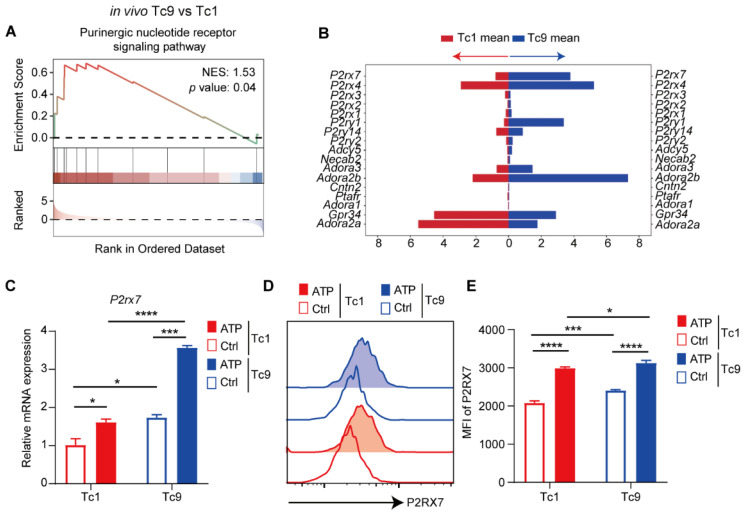
ATP promotes the expression of P2RX7 in Tc1 and Tc9 cells. (**A**) GSEA plot demonstrating a significant positive enrichment of the Purinergic Nucleotide Receptor Signaling Pathway gene set of Tc9 cells compared to Tc1 cells in vivo. (**B**) RNA-seq data showing differential mRNA expression of Purinergic Nucleotide Receptor Signaling Pathway gene set in endogenously sorted Tc1 and Tc9 cells. (**C**) Relative mRNA expression level of *P2rx7* gene in Tc1 and Tc9 cells cultured in vitro under control condition or treated with ATP (100 μM). (**D**) Flow cytometry histogram showing P2RX7 protein surface expression on Tc1 and Tc9 cells cultured under control or ATP-treated conditions (100 μM). (**E**) Quantification of the MFI of P2RX7 protein expression in each group (*n* = 3 biologically independent replicates, each derived from naive CD8^+^ T cells isolated from individual mouse spleens). Data are presented as mean ± SEM. * *p* < 0.05, *** *p* < 0.001, **** *p* < 0.0001.

Reanalysis of in vivo RNA-seq data revealed that purinergic nucleotide receptor signaling pathways were significantly enriched in Tc9 cells compared with Tc1 cells (Figure 5A). Notably, the key ATP receptor gene P2RX7, which has been implicated in ATP-mediated T cell regulation [32,33], was markedly upregulated in Tc9 cells (Figure 5B). This enrichment was validated by quantitative PCR, confirming higher baseline P2RX7 expression in Tc9 cells (Figure 5C). Importantly, ATP exposure further enhanced P2RX7 expression, suggesting the presence of a feed-forward amplification mechanism. Consistent results were observed at the protein level, where Tc9 cells displayed increased surface expression of P2RX7 (Figure 5D,E).

These findings indicate that Tc9 cells are intrinsically equipped with an enhanced purinergic signaling apparatus, enabling them to sense and transduce ATP stress signals more effectively. Rather than triggering apoptosis, ATP signaling in Tc9 cells appears to be rewired toward metabolic adaptation and TRM programming, thereby conferring a selective advantage within the high-ATP tumor microenvironment.

## 3. Discussion

Our study identifies extracellular ATP as a metabolic lineage selection signal during adoptive T cell therapy. Effective tumor destruction is intrinsically coupled to ATP accumulation within the tumor microenvironment, thereby generating a high-ATP niche that imposes selective pressure on infiltrating T cells. Rather than functioning solely as a cytotoxic stressor, ATP emerges as an instructive cue that differentially shapes the fate of transferred T cell subsets. Under these conditions, Tc9 cells exhibit a clear survival and functional advantage over conventional Tc1 cells.

Although both Tc1 and Tc9 cells encounter comparable ATP exposure following tumor killing, their responses diverge markedly. Tc1 cells are highly susceptible to ATP-induced apoptosis, whereas Tc9 cells display pronounced resistance to ATP-mediated cell death. This differential sensitivity provides a mechanistic explanation for the superior persistence of Tc9 cells in vivo [27,28,29]. Importantly, this phenomenon challenges the conventional view that high ATP uniformly suppresses T cell function and instead suggests that ATP responsiveness is lineage-dependent [20,33,34]. In addition, Tc9 cells exhibit improved mitochondrial function and enhanced forward TRM differentiation following ATP treatment.

Notably, the higher frequency of Tc9 cells observed in the blood, even outside the ATP-rich tumor microenvironment, reflects their intrinsically enhanced survival and persistence compared with Tc1 cells, which is consistent with our previous work and other published studies showing that Tc9 cells exhibit a less exhausted phenotype and superior longevity in vivo across multiple tumor models [27,28,35]. Moreover, Tc9 cells are generated in the presence of IL-4 and TGF-β (a well-established driver of TRM differentiation) [25]. This intrinsic differentiation condition predisposes Tc9 cells to a transcriptional and functional state that favors TRM differentiation, even before exposure to the tumor microenvironment. In the periphery, where repeated antigen stimulation is limited, Tc9 cells may retain a relatively less differentiated and longer-lived state. Upon entering the tumor microenvironment, sustained antigen stimulation together with high local TGF-β and extracellular ATP levels further promotes their differentiation into TRM-like cells and enhances their persistence—consistent with our in vitro repeated antigen stimulation model, where Tc9 cells differentiated into TRM-like cells much more readily than Tc1 cells, even after TGF-β withdrawal. And although we observed distinct metabolic advantages of Tc9 cells via Seahorse in vitro on day 10 post-stimulation, we did not compare the mitochondrial function parameters of the two cell subsets (Tc1 and Tc9) in vivo. Furthermore, we did not compare the ability of the two cell subsets to acquire a TRM phenotype under the same experimental conditions in vivo, which represents a limitation of our current experimental design. This lack of direct comparison hinders a more comprehensive understanding of the subset-specific functional differences in mitochondrial function and TRM formation potential in vivo between Tc1 and Tc9 cells, which will be addressed in future studies through targeted comparative analyses.

At the molecular level, Tc9 cells are intrinsically enriched in purinergic signaling pathways, including elevated expression of P2RX7, a major ATP receptor traditionally associated with ATP-induced cytotoxicity [20,33,34]. While high-level P2RX7 activation is known to promote pore formation and apoptosis, it is also implicated in regulating T cell differentiation and phenotypic remodeling. Our findings suggest that purinergic signaling in Tc9 cells is functionally rewired; instead of predominantly triggering cell death, ATP sensing promotes mitochondrial adaptation and TRM-directed programming. Nevertheless, the difference in P2RX7 surface staining between Tc1 and Tc9 under ATP treatment is modest, and this difference does not allow a definitive conclusion that P2RX7 is the sole mechanism driving Tc9 cells’ enhanced in vivo activity. Based on previous studies, P2RX7 may function in a context-dependent or transient manner, such that downstream signaling (e.g., Ca^2+^ influx) can be triggered without a marked or sustained increase in surface expression [36]. This indicates that the downstream consequences of ATP signaling depend not only on receptor expression but also on the intrinsic signaling architecture of the responding T cell subset. Nevertheless, in vivo experiments are still lacking to verify that ATP regulates mitochondrial function and TRM phenotype acquisition in Tc9 cells via P2RX7, although other study has shown that CD8^+^ TRM cells express high levels of the extracellular ATP receptor P2RX7 [37]. Furthermore, although P2RX7 appears to play a central role, other purinergic receptors such as members of the P2Y family may also be involved in regulating this adaptive response, which warrants further investigation.

Taken together, our results provide a potential mechanistic explanation for the superior antitumor activity of Tc9 cells reported in previous studies. In ATP-rich solid tumor environments characterized by continuous cell stress and necrosis, metabolically adaptable Tc9 cells are selectively stabilized, whereas ATP-sensitive subsets are preferentially eliminated. These findings suggest that purinergic signaling represent a tunable pathway for enhancing T cell persistence. Engineering T cells with enhanced ATP resilience or optimizing purinergic signaling may therefore represent promising strategies to improve the efficacy of ACT in solid tumors.

## 4. Materials and Methods

### 4.1. Mice and Cell Lines

Female C57BL/6J mice (6–8 week) were sourced from Laboratory Animal Center of Southern Medical University. OT-I (C57BL/6-Tg(TcraTcrb)1100Mjb/J) and CD45.1 congenic (B6.SJL-Ptprca Pepcb/BoyJ) mice were generously provided by Professor Bing Sun. Mice were housed in a specific pathogen-free (SPF) environment with controlled temperature of 20–26 °C, relative humidity of 40–70%, a 12 h light/12 h dark cycle, and provided with standard chow and filtered water ad libitum. The B16-OVA and MC38-OVA cell lines were routinely cultured and maintained in our laboratory.

### 4.2. Differentiation of Tc1 and Tc9 Cells

Total cells from the lymph nodes and spleen of mice were obtained in a sterile environment. Grind in a 70 μm cell filter using the push handle of a sterile syringe Following centrifugation, and red blood cells are lysed, the remaining leukocytes are washed twice with ice-cold PBS and resuspended with T cell medium (TCM). Naïve CD8^+^ T cells sorting were performed using MojoSort™ Naïve T Cell Isolation Kits (BioLegend, San Diego, CA, USA, Cat# 480040) according to the manufacturer’s instructions. Briefly, for every 1 × 10^7^ total cells, 10 μL of antibody cocktail is added and incubated on ice for 15 min, followed by the addition of 10 μL of pre-vortexed magnetic beads and a second 15-min incubation on ice. After adding appropriate volume of TCM, the cell suspension is transferred to a sterile FACS tube, placed in a magnetic separator within the laminar flow hood, and incubated for 5 min. The unbound fraction is carefully decanted into a new sterile FACS tube. The enriched cell pellet is then washed twice with cold PBS and resuspended in an appropriate volume of TCM. Tc1 cells were differentiated under the following conditions: culture in plates pre-coated with plate-bound anti-CD3 (clone 17A2, Bio X Cell, West Lebanon, NH, USA, Cat# BE0001-1, 7 µg/mL), supplemented with soluble anti-CD28 (clone 37.51, Bio X Cell, West Lebanon, NH, USA, Cat# BE0015-1, 1 μg/mL) and IL-2 (Novoprotein, Suzhou, China, Cat# C013, 100 U/mL). Tc9 cells were differentiated using plate-bound anti-CD3 (7 μg/mL), anti-CD28 (1 μg/mL), TGF-β1 (EPOTO Biotech, Nanjing, China, Cat# HF-2021, 1 ng/mL), IL-4 (Peprotech, Cranbury, NJ, USA, Cat# AF-214-14, 10 ng/mL), anti-IFN-γ (XMG1.2, Bio X cell, West Lebanon, NH, USA, Cat#BE0055, 20 μg/mL), and anti-IL-12 (C17.8, Bio X cell, West Lebanon, NH, USA, Cat# BE0051, 5 μg/mL). On the 5th day of induction, Tc1 and Tc9 cells were treated with 100 μM ATP or BzATP for 48 h, and then cells were harvested for experimental analysis.

For long-term expansion of both subsets, after 5 days of initial differentiation, the culture medium was replaced with T cell medium (TCM) containing IL-2 (100 U/mL) and maintained until day 8. On day 8, cells were restimulated for 4 h with TCM containing anti-CD3 (0.1 μg/mL), anti-CD28 (0.1 μg/mL), and IL-2 (100 U/mL). Following the stimulation, the cells were centrifuged, the stimulation medium was removed, and cultures were continued in fresh TCM supplemented with IL-2 (100 U/mL). TCM were maintained in RPMI 1640 medium supplemented with 10% FBS (ExCell, Suzhou, China, Cat# FSD500), 1× penicillin–streptomycin (Beyotime, Haimen, China, Cat# C0222), 1× L-glutamine (Gibco, Grand Island, NY, USA, Cat# 25030081), and 1× β-mercaptoethanol (Gibco, Grand Island, NY, USA, Cat# 21985023).

### 4.3. Tumor Inoculation and Therapy

C57BL/6J mice were subcutaneously injected with 5 × 10^5^ B16-OVA tumor cells. Five days after tumor inoculation, the Tc1 and Tc9 subsets of CD45.1^+^ OT-I mice were adopted via the tail vein in mice. Each mouse received more than 2 × 10^6^ of the respective CD8^+^ T cell subset along with 4 × 10^5^ dendritic cells (DCs). Tumor dimensions were measured every 3 days using a digital caliper. The longest diameter, (a) and the perpendicular short diameter, (b) were recorded, and tumor volume, (V) was calculated using the formula: V = 0.5 × a × b^2^. On day 7, peripheral blood was collected from the tail vein. On day 32, mice were euthanized, and terminal analyses were performed. The animal study protocol was approved by the Animal Care and Use Committee of Southern Medical University (Protocol: SMUL202502017).

### 4.4. Generation of Dendritic Cells (DCs)

On day 0, femurs and tibias were aseptically harvested from C57BL/6 mice. Bone marrow cells were flushed out using a 5 mL syringe and gently triturated three to four times in a 10 cm tissue culture dish to obtain a single-cell suspension. Red blood cells were lysed using RBC lysis buffer, and the remaining nucleated cells were counted; a minimum of 1 × 10^7^ viable cells per mouse was ensured for subsequent culture. Cells were resuspended in 20 mL of T cell medium (TCM) supplemented with 20 ng/mL murine granulocyte-macrophage colony-stimulating factor (GM-CSF) and seeded into T75 tissue culture flasks. Two days later, non-adherent cells were gently collected by swirling the flask and transferred to a new T75 flask containing fresh TCM supplemented with 20 ng/mL GM-CSF. Adherent cells in the original flask were discarded. On day 4, half of the medium in the culture flask was replaced with fresh TCM containing 20 ng/mL GM-CSF to support dendritic cell differentiation and expansion. On day 6, loosely adherent dendritic cells were harvested by gentle scraping, counted, and assessed for viability to ensure sufficient cell numbers for adoptive transfer experiments. Cells were then pulsed overnight with 2 μg/mL OVA257-264 peptide (SIINFEKL, Genscript, Nanjing, China, Cat# RP10611), 1 μg/mL lipopolysaccharide (LPS), and 20 ng/mL GM-CSF to induce maturation and antigen presentation. On day 7, mature dendritic cells were harvested by scraping, and viable cell counts were determined using trypan blue exclusion. These cells were subsequently used for adoptive T cell transfer experiments.

### 4.5. Flow Cytometry

Cells were harvested by centrifugation, and the culture supernatant was discarded. The cell pellet was washed once with FACS buffer and then transferred either to a 96-well V-bottom plate or a 1.5 mL tube. Cells were washed once with PBS, followed by resuspension in PBS-diluted Live/Dead dye. Cells were incubated on ice in the dark for 5 min to allow viability staining. After staining, cells were washed once with PBS and then twice with FACS buffer. Subsequently, surface antibody staining was performed by incubating the cells with fluorophore-conjugated antibodies on ice in the dark for 30 min. Following incubation, cells were centrifuged, the supernatant was removed, and the pellet was washed twice with FACS buffer. Finally, cells were resuspended in FACS buffer and transferred into flow cytometry tubes for immediate acquisition on a flow cytometer. Viability Dyes: eFluor™ 780 (eBioscience, San Diego, CA, USA, Cat# 65-0865-14). Surface markers: CD45 (BV510™, BioLegend, San Diego, CA, USA, Cat# 103138), CD45.1 (BV421™, BioLegend, San Diego, CA, USA, Cat# 110732), CD103 (APC, BioLegend, San Diego, CA, USA, Cat# 110905), CD69 (PE/Cy7, Thermo Fisher Scientific, Waltham, MA, USA, Cat# AF19854), CD8a (PerCP/Cyanine5.5, BioLegend, San Diego, CA, USA, Cat# 100734), Rabbit anti-p-SMAD2/3 primary antibody (Cell Signaling Technology, Danvers, MA, USA, Cat# 8828T), goat anti-rabbit secondary antibody (labeled Cy5, Abcam, Cambridge, UK, Cat# ab6564).

### 4.6. Mitochondrial Mass and Mitochondrial Membrane Potential Staining

To assess mitochondrial function, T cells were incubated in serum-free RPMI 1640 medium containing 50 nM MitoTracker Green (Thermo Fisher Scientific, Waltham, MA, USA, Cat# M7514) and 50 nM TMRM (Thermo Fisher Scientific, Waltham, MA, USA, Cat# I34361) at 37 °C in a CO_2_ incubator for 20 min. Following the staining incubation, the cells were washed twice with PBS and were then ready for subsequent surface staining.

### 4.7. Apoptosis Assay

Cell apoptosis detection was performed according to the kit manufacturer’s instructions (Biolegend, San Diego, CA, USA, Cat# 640914). The cells were washed with PBS and then resuspended in Annexin V Binding Buffer. Next, 5 μL of FITC-conjugated Annexin V was added to the cell suspension, and the cells were gently mixed. Subsequently, 5 μL of propidium iodide (PI) solution was added, and the mixture was again gently vortexed. The cells were incubated at room temperature in the dark for 25 min. Finally, 400 μL of Annexin V Binding Buffer was added to each flow cytometry tube, and the samples were immediately analyzed by flow cytometry.

### 4.8. Seahorse

Harvested and counted Tc1 or Tc9 cells on day 10 treated with ATP or not, after an initial 60-min incubation under standard conditions, the cells were transferred to a CO_2_-free incubator and allowed to equilibrate for an additional 30 min. OCR was then assessed using a Seahorse XF96 Analyzer (Agilent Technologies, Santa Clara, CA, USA) with sequential injections of mitochondrial modulators: oligomycin (1.5 μM), carbonyl cyanide-4-(trifluoromethoxy) phenylhydrazone (FCCP, 0.5 μM), and a combination of rotenone and antimycin A (0.5 μM each). All reagents were purchased from Agilent (Agilent Technologies, Santa Clara, CA, USA).

### 4.9. Quantitative Real-Time PCR (qPCR)

Total RNA was isolated from T cells using RNAex Pro RNA Reagent (AG, Hunan, China, Cat. No. AG21102). Complementary DNA (cDNA) was synthesized from 1 μg of total RNA using the Hifair^®^ III 1st Strand cDNA Synthesis SuperMix (YEASEN, Shanghai, China, Cat. No. 11137ES10). qPCR was performed on a QuantStudio™ Real-Time PCR System (Applied Biosystems, Thermo Fisher Scientific, Waltham, MA, USA) with SYBR qPCR Master Mix (Vazyme, Nanjing, China, Cat# Q711-02) and gene-specific primers synthesized by Tsingke Biological Technology (Beijing, China). GAPDH was used as an internal reference gene to normalize relative mRNA expression levels. Primer sequences are listed in Table 1.

### 4.10. RNA-Seq Data Analysis

RNA-seq data of tumor-infiltrating Tc1 and Tc9 cells from mice after adoptive cell transfer (ACT) were obtained from the Gene Expression Omnibus (GEO) database under accession number GSE176291, as described in our previous study [35]. The FPKM-normalized gene expression matrix was used for subsequent analyses, including fold change analysis, visualization of gene expression levels using heatmaps, and Gene Set Enrichment Analysis (GSEA). GSEA and pathway enrichment analyses were performed using the R package clusterProfiler (version 4.2.2), based on gene sets derived from the Gene Ontology (GO) and Kyoto Encyclopedia of Genes and Genomes (KEGG) databases. Visualization of GSEA results was implemented with the R package GseaVis (version 0.2.2). Heatmaps were generated using the R package pheatmap (version 1.0.12), and gene expression values were subjected to row normalization for better comparison across groups.

### 4.11. ATP Detection

Briefly, tumor tissues were isolated, weighed and gently washed with cold PBS, and dried with sterile filter paper before being placed on a 0.22 μm filter membrane fixed on a 15 mL centrifuge tube, and then centrifuged at 4 °C and 12,000 rpm for 10 min to obtain tumor interstitial fluid. ATP levels in tumor interstitial fluid or supernatant were quantified using the Enhanced ATP Assay Kit (Beyotime, Haimen, China, Cat# S0026) according to the manufacturer’s instructions. Luminescence value was measured using a microplate reader (BioTek, Winooski, VT, USA). The data were normalized to tumor weight to ensure comparability across samples.

### 4.12. Statistical Analysis

Experimental results were presented as mean ± standard error of the mean (SEM). Data were analyzed using GraphPad Prism 9 software. Statistical comparisons between two groups were performed using an unpaired Student’s *t*-test, while comparisons among three or more groups were assessed by one-way ANOVA followed by appropriate post hoc tests. Flow cytometry data were analyzed using FlowJo v10 software. Statistical significance was defined as: * *p* < 0.05, ** *p* < 0.01, *** *p* < 0.001, **** *p* < 0.0001, ns, not significant.

## 5. Conclusions

In conclusion, our study demonstrates that tumor-derived ATP plays a pivotal role in orchestrating T cell fate decisions during adoptive T cell therapy. We show that effective tumor control is accompanied by elevated intratumoral ATP release, which acts as a dual metabolic signal, simultaneously triggering T cell apoptosis and mitochondrial adaptive remodeling. Strikingly, we find that Tc9 cells exhibit a selective advantage in this high-ATP microenvironment, as they are resistant to ATP-induced apoptosis, display enhanced mitochondrial fitness, and are preferentially driven toward a TRM cell fate. This phenotypic plasticity is underpinned by the enrichment of purinergic signaling pathways in Tc9 cells.

Although these results support our core hypothesis that the ATP-TGF-β-TRM axis regulates Tc9 cell function, based on the small difference in P2RX7 surface staining observed after ATP treatment, P2RX7 may act through a context-dependent or transient signaling mechanism rather than a sustained upregulation of surface expression. The activation of P2RX7 can still trigger downstream signaling cascades [36], thereby driving the activation of the TGF-β pathway and TRM formation. Future studies are required to further validate the specific role of P2RX7 in this process and whether it mediates the characteristics of Tc9 cells compared to Tc1 cells, so as to strengthen the causal link between P2RX7 expression and the function of Tc9 cells in vivo.

Our findings delineate a novel paradigm where ATP-stress functions as a lineage selection signal, dictating T cell fate and therapeutic efficacy in ACT. These insights have profound implications for the design of more effective T cell-based cancer therapies, and suggest that targeting purinergic signaling may represent a promising strategy to enhance the persistence and anti-tumor function of adoptively transferred T cells.

## Figures and Tables

**Table 1:** Sequences of qPCR primers used in this study.

Gene	Forward Primer	Reverse Primer
*Itgae*	5′-GCAGAGAACCACAGGACGAA-3′	5′-TTCCGACTGGCTCAAACTCC-3′
*Runx3*	5′-CAGAGCCCCTTCCCACCATT-3′	5′-GGAAAGAACGCGAGAGCGTC-3′
*Tgfbr1*	5′-GGGGCGAAGGCAAGAAGTTT-3′	5′-ACAGAGGTGGCAGAAACACTG-3′
*Tgfbr2*	5′-TCCCAAGTCGGTTAACAGTGA-3′	5′-GTGAAGCCGTGGTAGGTGAG-3′
*Smad2*	5′-TGCTCCCTCCGTCTTCCGT-3′	5′-ATCCACACTCAGAAAAAGCCCG-3′
*Smad3*	5′-CCGTGCGGAAACCCAAACTT-3′	5′-ACTCTGGAGAACTTGCCCG-3′
*Smad4*	5′-GTGGCTTCCACAACTCCTGA-3′	5′-GGTCACTAAGGCACCTGACC-3′
*Gapdh*	5′-ATCAAGAAGGTGGTGAAGCA-3′	5′-GTCGCTGTTGAAGTCAGAGGA-3′

## Data Availability

The original contributions presented in this study are included in the article. Further inquiries can be directed to the corresponding authors.

## Supplementary Materials

The following supporting information can be downloaded at: https://www.mdpi.com/article/10.3390/ijms27073169/s1.
