# Supplementary material for: Extracellular ATP Functions as a Metabolic Lineage Selection Signal That Stabilizes Tc9 Cells During Adoptive T Cell Therapy
Source: Int J Mol Sci. 2026 Mar 31;27(7):3169. doi: 10.3390/ijms27073169 (PMC13072847; doi:10.3390/ijms27073169)
Supplement: Supplementary file 1 [file ijms-27-03169-s001.zip › ijms-4201896-supplementary.pdf]

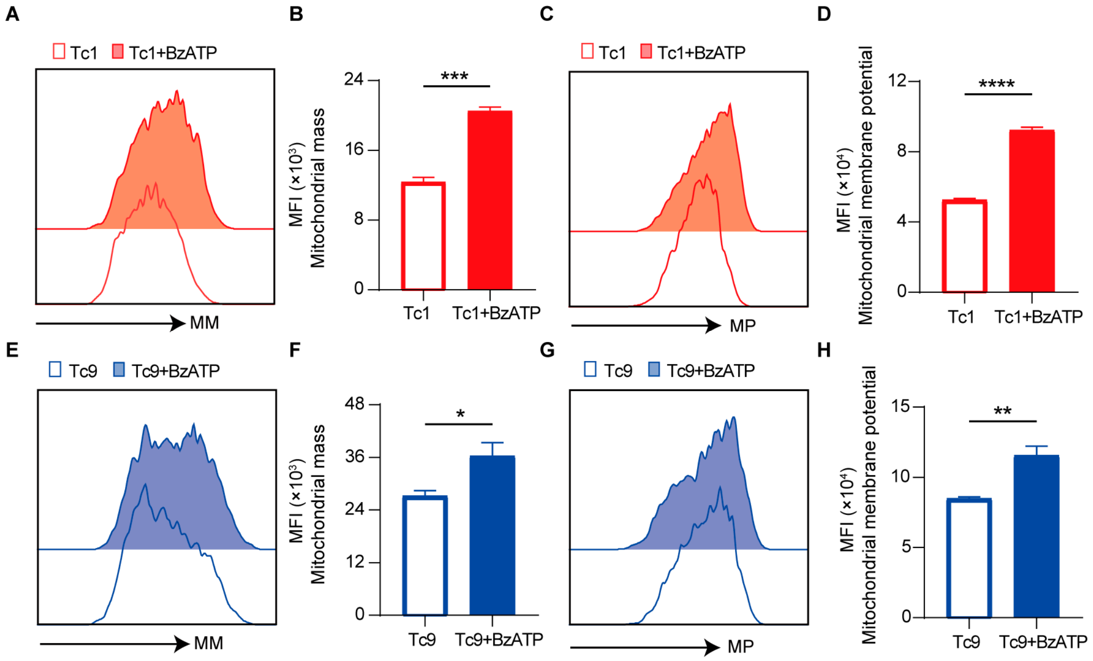

Figure S1. Mitochondrial metabolic remodeling in Tc1 and Tc9 cells under BzATP treatment.

(A) Representative plots showing the mean fluorescence intensity (MFI) of mitochondrial mass (MM, staining by MitoTracker) in Tc1 cells on day 5 treated with BzATP and untreated (Ctrl). (B) Quantification of the MFI of MM from each group. (C) Representative plots showing the MFI of mitochondrial membrane potential (MP, staining by TMRM) in Tc1 cells on day 5 treated with BzATP and Ctrl. (D) Quantification of the MFI of MP from each group. (E) Representative plots showing the MFI of MM in Tc9 cells on day 5 treated with BzATP and Ctrl. (F) Quantification of the MFI of MM from each group. (G) Representative plots showing the MFI of MP in Tc9 cells on day 5 treated with BzATP and Ctrl. (H) Quantification of the MFI of MP from each group. Data are mean ± SEM ($n = 3$). *$p < 0.05$, **$p < 0.01$, ***$p < 0.001$, ****$p < 0.0001$.
